# Filamentation in Atmospheric Air with Tunable 1100–2400 nm Near-Infrared Femtosecond Laser Source

**DOI:** 10.1038/s41598-019-48542-1

**Published:** 2019-08-19

**Authors:** Sean P. O’Connor, Christopher B. Marble, Dawson T. Nodurft, Gary D. Noojin, Adam R. Boretsky, Andrew W. Wharmby, Marlan O. Scully, Vladislav V. Yakovlev

**Affiliations:** 1 Texas A&M University, College Station, TX 77843 USA; 2 Engility Corporation, 4241 Woodcock Dr. Ste. B-100, San Antonio, TX 78228 USA; 3 Consortium Research Fellows Program, 4214 King Street, First Floor Alexandria, Virginia, 22302 USA; 4 711th Human Performance Wing, Airman Systems Directorate, Bioeffects Division, Optical Radiation Branch, 4141 Petroleum Rd., JBSA Fort Sam Houston, San Antonio, TX 78234 USA

**Keywords:** Near-infrared spectroscopy, High-harmonic generation

## Abstract

Intense femtosecond pulse filamentation in open-air has been utilized for long distance optical communication and remote sensing, but it results in nonlinear-effect driven eye hazards which are not addressed by current eye safety standards. A systematic study of filamentation in atmospheric air was performed using a tunable 100 fs near-infrared laser (1100 nm–2400 nm). While undergoing filamentation, each source wavelength was spectrally broadened resulting in supercontinuum and third harmonic generation in the visible and near-IR spectrum. We record the spectra at the center and fringes of the supercontinuum as it is imaged onto a planar surface. In a full beam collection regime, we report the energy of the sub-1000 nm light generation for source wavelengths from 1100 nm to 1600 nm and compare the energy density to the maximum permissible exposure values under the ANSI Z136.1 laser safety standard.

## Introduction

Laser induced filamentation is a nonlinear optical effect where intense laser pulses undergo self-focusing from the Kerr effect to overcome pulse diffraction and to propagate distances much larger than the diffraction length while maintaining a narrow beam diameter. Laser induced filamentation has been observed in solid, liquid, and gaseous media and has been studied in relation to high harmonic generation, supercontinuum generation, and as a method to generate single cycle and attosecond laser pulses^1–3^. Of particular interest is understanding open-air pulse propagation and atmospheric filamentation. Atmospheric filamentation provides applications such as atmospheric and remote sensing with techniques such as laser-induced breakdown spectroscopy^4^ and white light continuum ranging^5^. Atmospheric filamentation has also been studied as a method to achieve long distance optical communication and transmission of energy^6^.

Filamentation occurs when a high power, femtosecond laser pulse propagates through a medium and is self-focused (Kerr lensing) by the intensity dependent refractive index (n_2_). If the pulse power (Eq. (1)) exceeds a critical power threshold (Eq. (2)) the self-focusing will overcome the pulse diffraction causing the beam to collapse until multi-photon ionization occurs. The critical power threshold is dependent on the nonlinear refractive index (*n*_2_ ~ 5 × 10^−19^ *cm*^2^/*W* for air^7^) and a numerical factor (C) related to the beam shape (*C* ≈ 3.77 for a Gaussian beam, 3.72 < *C* < 6.4 in general^8^).

1 $$\begin{array}{c}{P}_{Exp}=K\frac{Energy}{Time},K=0.94\,{\rm{for}}\,{\rm{a}}\,{\rm{Gaussian}}\,{\rm{beam}}\end{array}$$

2 $$\begin{array}{c}{P}_{crit}=\frac{C{\lambda }^{2}}{8\pi {n}_{0}{n}_{2}}\end{array}$$

Plasma generation halts beam collapse. Defocusing from plasma generation balances self-focusing effects which results in the generation of one or more thin filaments of light which propagate distances that are orders of magnitude greater than the Rayleigh range without defocusing^6^. Laser filaments can display unusual properties such as pulse temporal self-compression. Self-compression during filamentation can reshape and shorten the time profile of the pulse leading to potential applications in attosecond pulse generation^3^.

Spectral broadening also accompanies filamentation through nonlinear effects such as self-phase modulation and self-steepening which modulates the initial pulse’s spectrum into a supercontinuum composed of a broad range of wavelengths. Supercontinuum generation was first reported in 1970 in which 530 nm picosecond pulses were propagated through bulk BK7, and the resulting pulse was spectrally broadened to a 400–700 nm spectral range^2^. Since then, intense “white-light” continuums generated in filamentation and supercontinuum studies have found a broad spectra of applications including optical coherence tomography^9^, colorimetry^10^, STED microscopy^11^, pump-probe spectroscopy^12,13^, and Raman spectroscopy^14–17^.

The first observation of laser filamentation in air was by Braun *et al*. with a 775 nm input pulse^18^. Since then, atmospheric filamentation has been reported at many wavelengths shorter than 1030 nm and in the mid-IR at 3.9 µm (see Table 1). Filamentation with a near-IR input beam has been demonstrated in highly-pressurized gases^19–22^. To our knowledge, filamentation in atmospheric air with a tunable near-IR laser source has not been attempted.

**Table 1:** Filaments in air observed in literature.

Wavelength (nm)	FWHM Time Duration (fs)	Pulse Energies (mJ)	Critical Power (C = 3.77) (GW)	Critical Power (C = 6.4) (GW)	Experimental Pulse Powers (GW)	Reference
248	450	2–5	0.2	0.3	4–10	^31^
248	5000	7.5	0.2	0.3	1.4	^31^
400	~60	2.3	0.5	0.8	36	^7^
406	40	1	0.5	0.8	7	^32^
527	900	1.6–4	0.8	1.4	2–4	^33^
775	200	2–50	1.8	3.1	9–235	^18^
800	42	2.3	1.9	3.3	51	^7^
1030	200	1.5–10	3.2	5.4	7–47	^34^
3900	~80–200	20–30	46	77	200–350	^8^
10200	3500	5200	310	530	1400	^35^

Critical powers are estimated with Eq. (2) using *n*_2_ ~ 5 × 10^−19^ *cm*^2^*/W* as an estimate from literature^7^, and experimental pulse powers are computed with Eq. (1) assuming a Gaussian pulse.

Despite the use of open-air high-power lasers, the eye safety implications of supercontinuum generation in air have not been studied. Eye safety standards such as ANSI Z136.1 set maximum permissible exposure (MPE) limits based on the laser wavelength and time duration, but they do not account for nonlinear processes altering the laser wavelength^23^. Concurrent studies of high harmonic generation in zinc selenide have shown the generation of sufficient visible and near-infrared radiation light from mid-IR source wavelengths to pose a retinal hazard by ANSI standards^24^. Eye safety standards inform the choice of laser eye protection (LEP) used by laser operators who choose LEP that absorbs or reflects the laser wavelength with the minimum reduction of visual acuity and color perception. Filamentation and supercontinuum generation raise the risk of new or additional eye safety hazards being generated during laser operation at wavelengths outside the operating laser wavelength^25^. If LEP is chosen that does not also provide adequate protection against the constituent wavelengths of the supercontinuum, a retinal injury could occur. One possible eye hazard is the production of sub-1050 nm wavelengths which have lower exposure limits compared to >1200 nm pulses since longer wavelength pulses (particularly >1400 nm pulses) are strongly absorbed in the aqueous and vitreous humors of the eye prior to reaching the retina^23^.

We demonstrate filamentation in atmospheric air in the lab with tunable near-infrared laser pulses with wavelengths ranging from 1100 nm to 2400 nm. We report the spectrum of the resulting supercontinuum generation as it is projected onto a sheet of paper by measuring the center and fringes of its output for different source wavelengths in increments of 100 nm. The input energy for each source wavelength is measured prior to filamentation. The experimental setup is then modified to collect and record the energy of the visible component of the pulse. We measure the output energy in the sub-1000 nm regime following filamentation for the source wavelengths 1100 nm to 1600 nm. We show that filamentation of the input pulse results in intense visible light generation due to both harmonic generation and supercontinuum generation. In some instances, the visible light generation exceeded the MPE values set by the ANSI laser eye safety standard confirming that near-IR pulse filamentation can generate visible eye hazards.

## Methods

**Figure 1:**
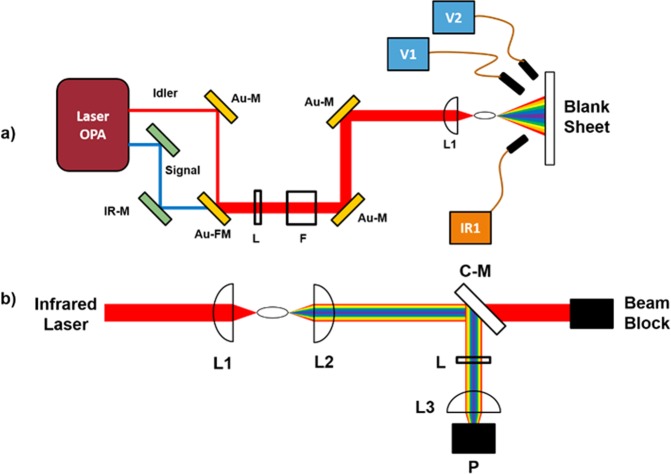
(**a**) The experimental setup to observe the supercontinuum generation. (**b**) Removed blank sheet for spectral collection and added optics for visible light collection and power measurement after supercontinuum generation. Au-FM: Gold flip mirror. Au-M: Gold mirror. C-M: Cold Mirror. F: Filter wheel. IR-M: Infrared mirrors. L: Long-pass filter. L1, L2, L3: Converging lenses. P: Power meter. V1, V2: Visible spectrum spectrometers. IR1: Infrared spectrum spectrometer.

Nominally 100 fs near-IR pulses with a source wavelength ranging from 1100 nm to 2700 nm were produced using a High Energy TOPAS optical parametric amplifier (OPA, model TH8F1) and a Spectra Physics^®^ Spitfire Ace regenerative amplifier (model 8PTFPA-100F-1K-ACE) following the design of previous studies^26,27^. Pulses from 1100 nm to 1600 nm were generated using the OPA’s signal beam, and pulses from 1600 nm to 2700 nm were generated using the OPA’s idler beam. A flip mirror (labeled Au-FM in Fig. 1(a)) was used to select either the signal or idler beam as the filamentation source, and the beam diameter ranged from 6 mm to 9 mm depending on the selected wavelength. The beam passed through a 1000 nm long pass filter (Thorlabs^®^ FGL-1000, labeled L in Fig. 1(a)) to eliminate extraneous visible light that was emitted from the OPA. A collection of neutral density filters (labeled F in Fig. 1(a)) attenuated the beam energy, and the beam was focused in air using a +75 mm calcium fluoride converging lens (labeled L1 in Fig. 1(a)) which resulted in filamentation. The chirp from quadratic and cubic dispersion due to the optics was computed^28^. The quadratic dispersion (linear chirp) was found to be positive, but negligible for all wavelengths. The cubic dispersion from the neutral density filters was found to be nonnegligible (and negative) only for 1100 nm and 1200 nm measurements. The supercontinuum generated during filamentation was projected onto a blank sheet of paper. For each wavelength, the supercontinuum spectra were recorded for three pulse energies using three spectrometers. Two spectrometers were pointed at the center of the supercontinuum, one recording the visible spectrum (V1), an Ocean Optics^®^ USB 2000+, and one recording the infrared spectrum (IR1), an Ocean Optics^®^ NIR-512. A second Ocean Optics^®^ USB 2000+ spectrometer was used to record the visible fringe of the supercontinuum (V2).

The experimental setup was then modified into a collection regime so that the visible light generated during filamentation of the signal beam was focused down and collected (see Fig. 1(b)). A +200 mm focusing lens was used to collimate the center of the supercontinuum pulse, and the collimated beam was reflected off a cold mirror (labeled C-M in Fig. 1(b)) to remove the near-infrared component of the beam. The reflected light was measured using a power meter (Coherent^®^ PM10, P in Fig. 1(b)) with and without a 1000 nm long pass filter (Thorlabs^®^ FGL-1000) in the path of the reflected beam. A 1-inch diameter, BK7 lens (labeled L3 in Fig. 1(b)) decreased beam width to fit the sub-1000 nm light onto the power meter. After taking measurements for all source beam wavelengths from 1100 nm to 1600 nm in increments of 100 nm, the +200 mm collection lens was replaced with a +100 mm collection lens and the measurements of reflected light with and without the long pass filter were repeated. To account for the absorption of infrared light due to the 1000 nm long-pass filter, the absorption spectra for the long pass filter from 200 nm to 1600 nm was measured using a spectrophotometer (Cary 6000i, Agilent^®^). We calculated the energy of the sub-1000 nm component of the output pulse and the conversion efficiency of filamentation after accounting for the absorption due to the long pass filter.

The relative efficiencies at each wavelength were determined for the NIR-512 and USB 2000+ spectrometers using the LS-1 Tungsten Halogen lamp light source (Ocean Optics^®^). For each spectrometer, the LS-1 was coupled to the spectrometer using the same fiber used in the filamentation experiment and the LS-1 spectrum was recorded. The relative efficiencies were then calculated by comparing the experimentally measured LS-1 spectra against the expected LS-1 spectra of a 3100 K blackbody. The relative efficiencies were then used to correct the intensity response from our recorded spectral data.

## Results and Discussion

**Figure 2:**
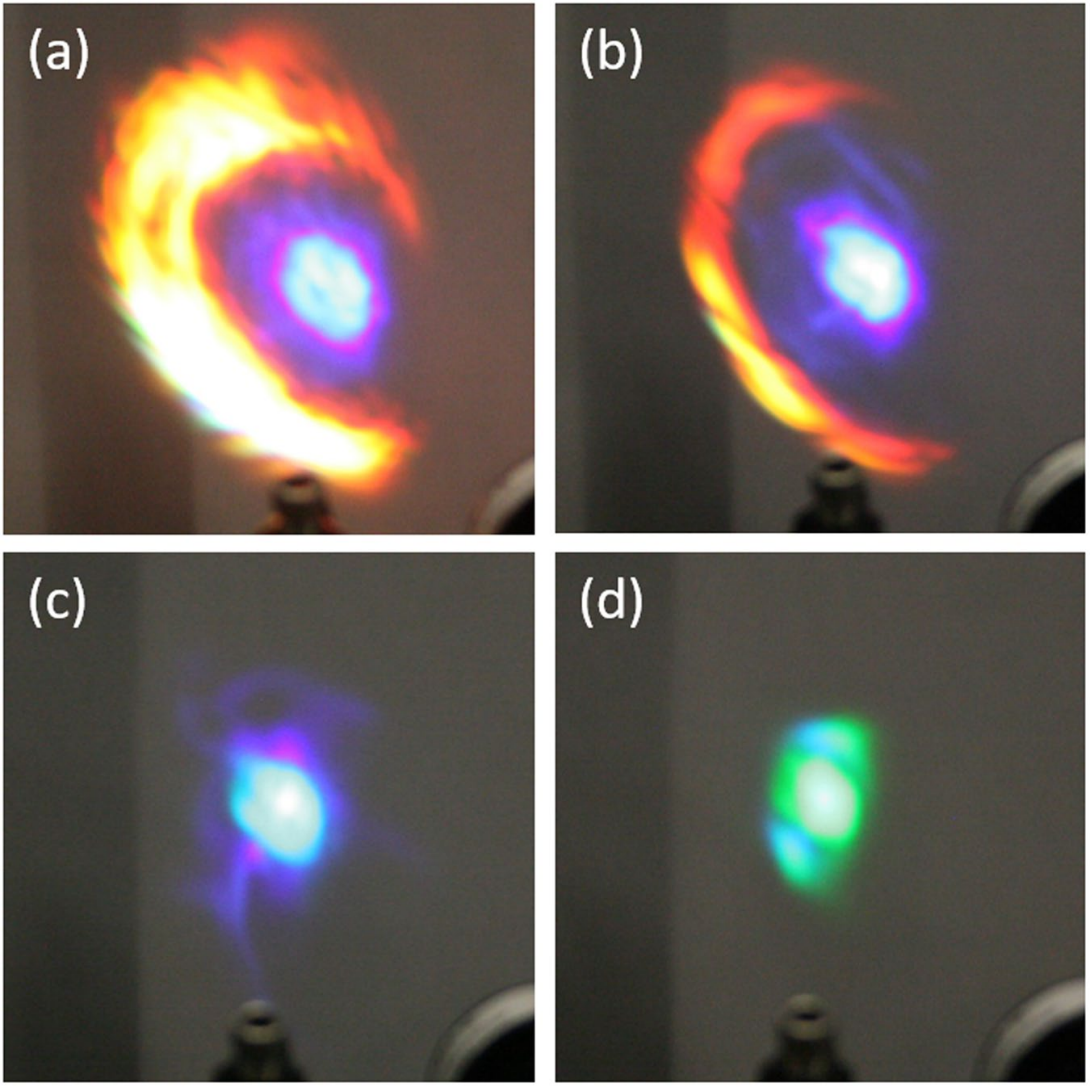
Supercontinuum generation imaged onto a sheet of blank white paper. Source wavelength for each continuum is (**a**) 1300 nm (**b**) 1400 nm (**c**) 1500 nm (**d**) 1600 nm.

Using the setup in Fig. 1, filamentation was achieved for wavelengths ranging from 1100 nm to 2400 nm. For each wavelength, the supercontinuum projected onto the paper was photographed (see Fig. 2). In addition to photographing the spectra, the visible and near-IR spectra were recorded by three spectrometers as shown in Fig. 1(a). From visual examination and spectrometer measurements, the center of the supercontinuum was observed to consist of the fundamental infrared wavelength and third harmonic light. Consistent with previous explorations of conical emission from supercontinuum generation in air, the fringes of the supercontinuum were observed to consist of supercontinuum broadened light with larger conic emission angles corresponding to shorter (visible) wavelengths^29,30^. In this experiment, the fringes were generally less intense than the third harmonic light, with the exception of the measurements taken with source wavelengths at 1300 nm and 1400 nm (see Fig. 2(a,b)).

The measured wavelengths for the third harmonic peaks are reported in Table 2, along with the predicted third harmonic wavelength for each source wavelength. We find that our wavelength measurements for the observed third harmonics were within 6% of the predicted wavelength values. We also report the supercontinuum spectral ranges we measured from each spectrometer in Table 2. Beam diameters for the 1100 nm and 2400 nm sources were not acquired as the maximum pulse energy was too weak to detect with the beam camera.

**Table 2:** Beam parameters for spectra generation.

Wavelength (nm)	Max Pulse Energy (µJ)	Beam Diameter (mm)	Supercontinuum Range 1 (nm)	Supercontinuum Range 2 (nm)	Predicted 3^rd^ Harmonic (nm)	Observed 3^rd^ Harmonic (nm)
1100	210 ± 30	NM	1030–1130	NO	367	352
1200	740 ± 60	7.5	980–1230	615–850	400	389
1300	840 ± 60	7.6	1015–1355	660–870	433	431
1400	1530 ± 20	6.9	1145–1430	595–860	467	464
1500	1360 ± 60	7.0	1285–1575	NO	500	498
1600	1180 ± 20	6.5	1475–1675	NO	533	530
1600-I	1220 ± 20	7.3	1200–1685	670–870	533	529
1700-I	1420 ± 40	8.7	1220–1720	545–840	567	559
1800-I	1025 ± 6	8.4	1445–1705	655–870	600	604
1900-I	1185 ± 8	9.8	1520–1720	NO	633	635
2000-I	970 ± 7	9.1	1565–1715	NO	667	681
2150-I	885 ± 4	8.3	NO	NO	717	757
2200-I	980 ± 20	8.5	NO	NO	733	734
2300-I	684 ± 8	7.8	NO	NO	767	784
2400-I	47 ± 4	NM	NO	NO	800	813

Wavelengths followed by “-I” were generated using the Idler beam. Acronyms: Not Measurable (NM), Not Observed (NO).

**Figure 3:**
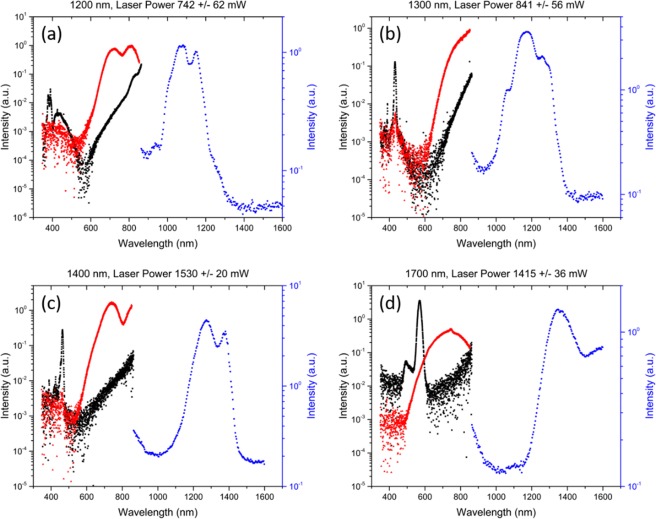
Supercontinuum spectra of the center (black) and fringes (red) of the visible output as well as the center output of the IR (blue) are shown above. The left axis corresponds to the visible light data, and the right axis corresponds to infrared data. Source wavelengths shown are (**a**) 1200 nm (**b**) 1300 nm (**c**) 1400 nm (**d**) 1700 nm. Intensities shown were corrected using the relative efficiencies from the LS-1 light source.

Spectra measured from the blank sheet of paper are shown in Fig. 3. We observed supercontinuum generation in the visible and in the infrared regimes, and this light was at least an order of magnitude greater than the noise floor in Fig. 3. Distinguishable third harmonic generation peaks can be seen on each figure at the wavelengths that are listed in Table 2. We observed spectral broadening of the pump pulse’s wavelength along with the development of a spectral lobe similar to previous studies in highly pressurized gas^20–22^. We find that the spectral lobe is blue-shifted with respect to the pump pulse’s central wavelength.

**Figure 4:**
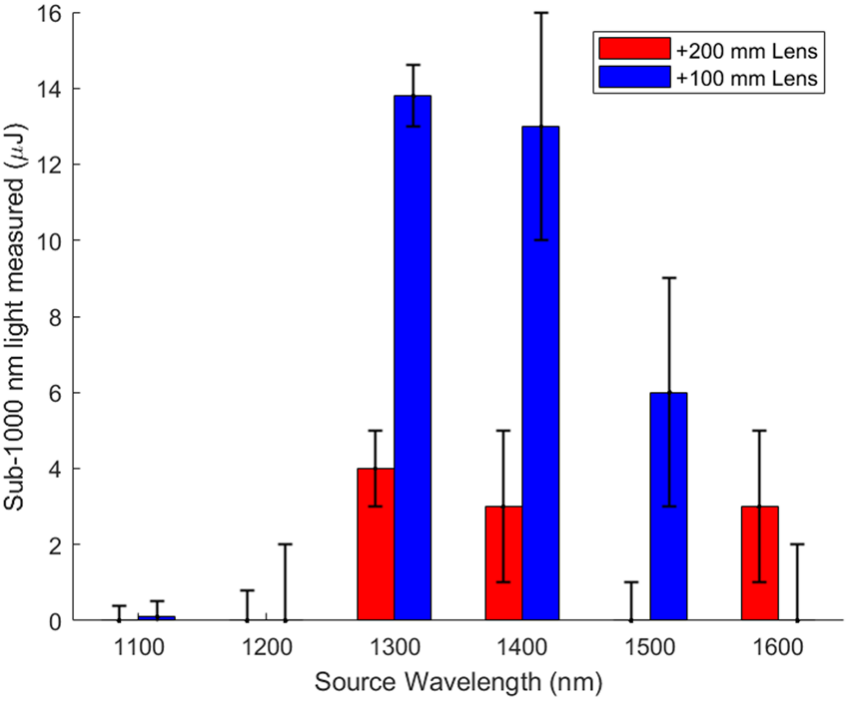
Sub-1000 nm light generation collected from power meter (P) in the experimental setup in Fig. 1(b). Input pulse energy for each source wavelength is the max pulse energy listed in Table 2. The starting beam diameter of each pulse is also listed in Table 2.

Having recorded the spectra of the supercontinuum generation, we then measured the pulse energy using the setup in Fig. 1(b) with and without the FGL-1000 long pass filter. By measuring the energy with and without the long pass filter and correcting for the infrared wavelength loss of the long pass filter, the sub-1000 nm pulse energy was determined for the +200 mm lens setup and +100 mm lens setup (Fig. 4). In both configurations, 1300 nm and 1400 nm wavelengths were observed to generate micro-Joules of sub-1000 nm radiation, which is a clear eye hazard based on the ANSI limits and input beam radius. If the 1300 nm beam was recollimated to a beam diameter of 7.6 mm, the sub-1000 nm light energy per unit area would approach (23.9 ± 1.4) µJ/cm^2^, exceeding the ANSI limit of 0.1 µJ/cm^2^ by two orders of magnitude^23^. These results agree with visual observations of the supercontinuum at 1300 nm and 1400 nm which were intense even when scattered from a piece of paper (Fig. 2(a,b)). The +200 mm experiment suggests that the 1600 nm filament was a visible eye hazard while the +100 nm lens setup suggested that the 1500 nm filament was a visible eye hazard. Both setups failed to collect the entire supercontinuum spectra. The +200 mm lens was only able to capture the center, third harmonic region, of the continuum resulting in lower reported pulse energies.

## Conclusion

We performed a systematic study of near-IR filamentation in atmospheric air using femtosecond pulses with source wavelengths ranging from 1100–2400 nm. We demonstrated that these near-IR wavelengths were able to generate a supercontinuum in the visible spectrum, as well as distinct third harmonics. Measurements of the spectrum exhibited a strong third harmonic response that appears within the visible light spectrum for most of our input wavelengths, with the exception of near-IR third harmonics for >2000 nm source wavelengths.

These third harmonic peaks in the visible light spectrum demonstrate a clear laser-eye safety hazard according to the ANSI guidelines^23^. The generation of sub-1050 nm infrared third harmonic light for >2000 nm source wavelengths is worrisome from an eye safety perspective, because laser operators would observe an intense, visible pulse for shorter source wavelengths. However, they would not be aware of their exposure to sub-1050 nm infrared harmonics and continuum for >2000 nm wavelengths. The supercontinuum and high harmonic generation from the ultrashort pulses need to be taken into consideration in applications of atmospheric filamentation. In particular, the infrared laser eye protection worn to protect against exposure to the source wavelength may not provide sufficient protection against the third harmonic wavelength.

Further study of near-IR filamentation is necessary to determine the hazard that is posed from supercontinuum and harmonic generation in the infrared regime, because the 1000–1200 nm infrared energies were not recorded in the collection setup (Fig. 1(b)) of our study. For some of our source wavelengths, we observed that supercontinuum generation occurs in the near-IR regime which can propagate to the back of the retina, and we note that >2000 nm pulses generate spectra located almost entirely in the near-IR. Quantification of this 1000–1200 nm near-IR generated light is essential to understanding the risk to laser-eye safety that atmospheric air pulse propagation and filamentation can pose. Furthermore, the strong nonlinearity of the supercontinuum and harmonic generations in atmospheric air propagation indicate that the resulting spectral output is subject to large variations from changing the experimental parameters, such as the focal length and atmospheric conditions. Future work characterizing sub-1050 nm generation efficiencies from nonlinear processes with fine control of source pump energy and examining a worst-case analysis will be necessary to provide specific protection recommendations for laser-eye safety.
